# Arterial interventional chemotherapy and IMRT with concurrent chemotherapy for nasopharyngeal carcinoma with intracranial involvement

**DOI:** 10.3892/ol.2013.1407

**Published:** 2013-06-17

**Authors:** XINGLAI FEN, WEIFENG QIN, WUAN BAO, FENG JIANG, BIN LI, FUJUN HU, XIAOZHONG CHEN

**Affiliations:** Radiation Therapy Department of Oncology, Zhejiang Cancer Hospital, Hangzhou, Zhejiang 310022, P.R. China

**Keywords:** nasopharyngeal carcinoma, comprehensive treatment, interventional chemotherapy, intensity modulated radiation therapy

## Abstract

The aim of this study was to ensure a high dose of intensity modulated radiation therapy (IMRT) was delivered to tumor tissue with a low dose to normal organs. Seldinger interventional techniques were used to inject chemotherapy drugs for nasopharyngeal carcinoma (NPC). IMRT was conducted 3 weeks after intervention. Primary tumor volume was reduced by 42.76% after 2 doses of interventional chemotherapy and intracranial tumor volume was reduced by 55.63%. All patients presented grade II and above nasopharyngeal mucositis. In the 2 years following radiotherapy, overall survival (OS) was 83.3% and progression-free survival (PFS) was 75%. In conclusion, T4 NPC patients with intracranial extension received induction chemotherapy followed by IMRT and concurrent chemotherapy, which proved to be efficacious and well tolerated.

## Introduction

Nasopharyngeal carcinoma (NPC) is one of the most common malignant tumors, with its incidence rate ranking first among head and neck cancers. In China, more than 90% of NPC cases are pathological type III according to the World Health Organisation (WHO) criteria, and are sensitive to radiation. Therefore, for patients who are newly diagnosed and without metastasis, radiotherapy is the preferred treatment (1). In the last decade, the effect of NPC radiotherapy has been significantly improved. The local control rate of early NPC is 70–90%, while that of T3-4 NPC is 50% (2).

Intensity modulated radiation therapy (IMRT) is a breakthrough in radiation oncology. It delivers a highly concentrated dose to the target area to kill tumor cells, while adjacent healthy tissue is unaffected, which increases the gain ratio of radiotherapy. The local control rate of T1-3 patients is >90%, but that of T4 patients is only ∼85%. In certain T4 NPC patients with intracranial extension, the space between the tumor and surrounding organs is small, so the radiation dose must be limited in order to protect the normal tissues and organs. This can result in local control failure and affect the survival rate (3).

In order to improve the local control rate and reduce distant metastasis, many clinical studies of treatment of locally advanced NPC with radical radiotherapy and chemotherapy have been performed. Since the results of intergroup 0099 trial were published (4,5), the treatment strategy of NPC has been modified significantly and concurrent chemoradiotherapy has been used as the standard treatment of locally advanced NPC. Induction plus concurrent chemoradiotherapy is now standard in the treatment of NPC. A clinical phase II study has shown promising results with improved distant metastasis-free and overall survival; the choice of chemotherapy drugs, delivery methods and course number, however, needs further research. The European Society for Medical Oncology (ESMO) NPC clinical guidelines (6) suggest that while chemotherapy is not the standard treatment for NPC, it improved disease-free survival, suggesting that it may be used for the treatment of locally advanced NPC.

Arterial infusion chemotherapy has the features of high local drug concentration and a stronger effect of local tumor toxicity with low systemic side effects. It is used in the treatment of a variety of tumors.

The purpose of this study was to investigate the treatment of NPC patients with intracranial extension with arterial infusion chemotherapy. The aim was to reduce the volume of the primary tumor and expand the spatial distance between the tumor and vital organs. This will improve the dose distribution of IMRT and meet the target dose requirements while protecting the brain stem and nerve tissues. It is expected that by improving local treatment, overall survival (OS) time will also be improved. The aim was to find a combination providing optimal treatment for locally advanced NPC.

## Materials and methods

### General information

Twelve cases of NPC were selected between March and December 2009. All patients signed informed consent before treatment. The study was approved by the Ethics Committee of Zhejiang Cancer Hospital, Hangzhou, China. Pathologically all types were WHO III, 11 cases were males and 1 case was female. Aged from 38 to 68 years, the average age was 52.7 years. The clinical stages of all cases demonstrated cavernous sinus and intracranial invasion indicating UICC 2002 staging T4N0-2M0IVa; the Karnofsky values were ≥80. No patients had serious liver and kidney function, heart function and coagulation disorders or cerebral hemorrhage or infarction history.

### Methods

Seldinger interventional techniques were used. Briefly, the patients were incubated percutaneous femoral artery puncture, catheterization of bilateral carotid artery, then angiography; next inserted into maxillary artery, ascending pharyngeal artery and branch of the target lesions. Chemotherapy drugs (80 mg/m^2^ cisplatin and 60 mg/m^2^ epirubicin) were injected the day after interventional therapy and 2,500 mg/m^2^ 5-FU was administered using a chemotherapy electronic pump for 120 h. There were 21 days per cycle. Two interventional treatments were conducted prior to radio-therapy. IMRT was conducted 3 weeks after intervention. During radiotherapy, two cycles of synchronous single-agent chemotherapy were conducted (80 mg/m^2^) (Tables I and II).

### Observation methods and clinical evaluation

All patients gave routine blood samples and their liver and kidney function was tested every week before and during treatment. During treatment, tumor regression, general condition, mucous membrane and gastrointestinal reactions were checked and recorded. Nasopharyngoscopy and nasopharynx enhanced MR was performed before treatment, two weeks after the second intervention, at the end of radiotherapy, and 3 and 6 months after radiotherapy. Efficacy was evaluated according to the measurement standard of the WHO, as follows: Complete remission (CR): tumors disappear completely, the disappearance time is not less then 4 weeks; partial remission (PR): tumors shrink by more than 50%, the remission time is 4 weeks or more; no change (NC): tumor shrinkage does not exceed 50% or increase does not exceed 25%; tumor progression (PD): tumor increase >25%. Toxicity was evaluated according to the anticancer drug toxicity standard (CTC3.0).

## Results

### Changes in tumor blood flow

**Figure 1.**
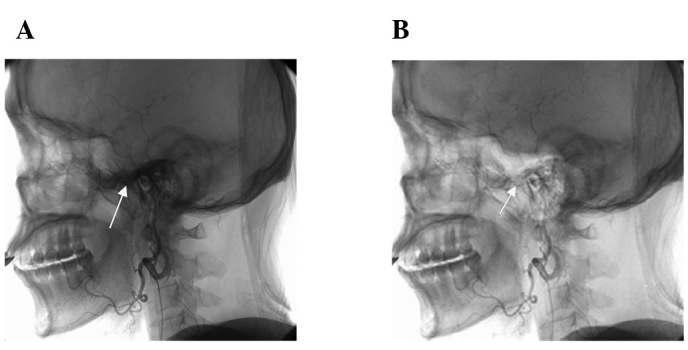
Changes in tumor blood supply. (A) Tumor blood vessel density in pars nasalis pharyngis is higher after first interventional therapy. (B) Tumor blood vessel density in pars nasalis pharyngis is reduced markedly after second interventional therapy.

The 12 patients completed two interventional treatments. Digital subtraction angiography showed that the internal blood network of NPC reduced following interventional treatment at different degrees (Fig. 1).

### Changes in tumor volume

**Figure 2.**
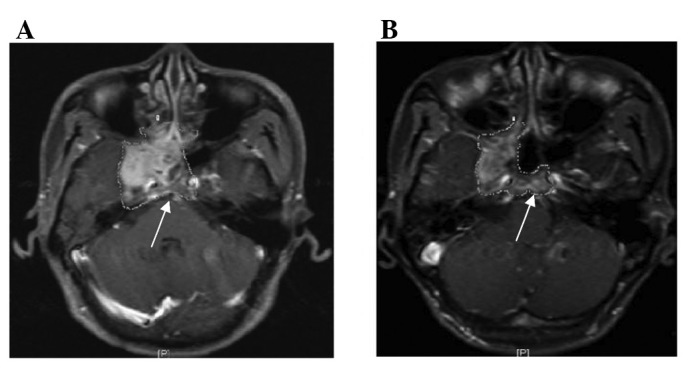
The tumor volume change. (A) The tumor volume is larger before interventional therapy. (B) The tumor volume is reduced after two interventional therapies.

The average tumor volume of the 12 patients was decreased from 45,730 to 26,174 mm^3^ after the second interventional chemotherapy, the average reduction was 42.76%; the tumor intracranial total volume was reduced from 9,268 to 4,112 mm^3^, the average reduction was 55.63%. The distance between the tumor and the brain stem, optic nerve and optic chiasm were expanded to 3.5, 5.1 and 5.8 mm from 2.3, 4.4 and 4.6 mm, respectively (Fig. 2 and Table III). In order to meet the IMRT dose, 95% PTV must meet the dose of the target. The dose changes before and after intervention are shown in Table V.

### Evaluation of tumors

At the end of radiotherapy the tumor situation was as follows: 6 CR cases, 5 PR cases and 1 PD case (distant metastasis appeared during radiotherapy, so treatment was changed to local palliative irradiation and systemic chemotherapy).

### Follow-up

**Figure 4.**
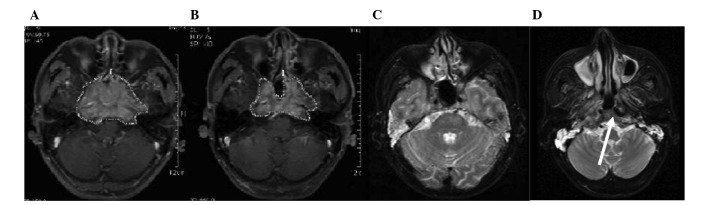
The follow-up outcome. (A) MR before therapy. (B) MR after two interventional chemotherapy treatments. (C) CR imaging after radiotherapy. (D) A deep ulcer appeared after 3 months of therapy.

Three months after radiotherapy, the patients were followed up and 7 cases of CR, 3 cases of PR, 1 case of PD and 1 case of CR were found. One patient succumbed to nasopharyngeal ulcer hemorrhage. The nasopharynx and intracranial have a large tumor volume, tumor regression after interventional chemotherapy, nasopharyngeal mucosa reached grade IV at the end of radiotherapy and the local secondary infection failed to control, the ulcers invaded neck blood vessels and caused hemorrhage causing mortality (Fig. 4). The patients were followed up 2 years later. The OS and progression-free survival (PFS) rates were 83.3 and 75%, respectively. One patient succumbed to nasopharyngeal ulcer hemorrhage, one succumbed to distant metastasis to multiple organs, and one patient recurred. However, there was no spinal cord, brain tissue or cranial nerve damage in any of the cases.

## Discussion

IMRT is a revolution in radiation oncology technology, which has significantly improved treatment for NPC compared with conventional radiotherapy, although it still has a higher risk for T4 patients (7), particularly for T4 NPC patients with intracranial extension, whose tumor and vital organs (brain stem, optic nerve, optic chiasm and temporal lobe) are closely connected. Limiting the radiation dose protects the normal tissues and organs but may result in an insufficient dose to treat the tumor and lead to local control failure. Conversely, if the dose is too high it may lead to serious brain, spinal cord and neurological complications in the surrounding healthy organs (8). An insufficient dose to the tumor or excessive dose to normal tissue is a major obstacle for radiotherapy doctors. Induction radiotherapy plus concurrent chemotherapy has been shown to improve OS and local control rates of NPC (9–12). Interventional chemotherapy has become one of the treatments of a variety of tumors with the development of interventional techniques. Drugs injected directly into the tumor nutrient vessels with interventional chemotherapy increase the local concentration several times more than drugs injected systemically (13). Selective arterial chemotherapy can destroy a large number of cancer cells in a short period, which not only shrinks the tumor volume, reduces the hypoxic cells and improves the sensitivity of radiotherapy, but also expands the space between the tumor and other organs to implement IMRT radiotherapy.

This study showed that the primary tumor volume was reduced by 42.76% following two doses of interventional chemotherapy, and the intracranial tumor volume was reduced by 55.63%. The distance from the brain stem, optic nerve and optic chiasm were increased by 1.2, 0.7 and 1.2 mm, respectively. This resulted in a smaller GTVnx target. By ensuring the sufficient therapeutic dose to the tumor, the dosage in the brain stem, optic chiasm, optic nerve and temporal lobe were decreased by 15.26, 11.11, 13.55 and 8.95%.

The patients tolerated treatment well and had no serious grade IV adverse reactions to stop treatment. Compared to another study on conventional PF (DDP + 5-Fu) systemic induction chemotherapy (14), the patients did not have hematological toxicity, gastrointestinal tract, liver or kidney damage. At the end of radiotherapy, there were 6 cases of tumor CR (50%) and 11 cases of CR plus PR (91.6%). Two years after radiotherapy, OS was 83.3% and PFS was 75%. After interventional chemotherapy, all patients presented grade II and above nasopharyngeal mucositis, 7 cases of grade III (58.3%) and 1 case of grade IV (8.3%). One patient succumbed to nasopharyngeal ulcer hemorrhage, tumor shrinkage in nasopharynx and intracranial after interventional chemotherapy was evidient, and nasopharyngeal mucosa appeared grade IV at the end of radiotherapy. Local secondary infection failed to control, the ulcers invaded neck blood vessels and caused hemorrhage and mortality.

According to research, T4 NPC patients with intracranial extension were given induction chemotherapy followed by IMRT plus concurrent chemotherapy with good success. This treatment method controlled tumors and protected the surrounding tissues and organs, which is a good choice for locally advanced T4 NPC patients. The OS and PFS were acceptable according to the 2-year follow-up. However, to help reduce the distant metastasis and local recurrence, long-term observation is required.

## Figures and Tables

**Figure 3.**
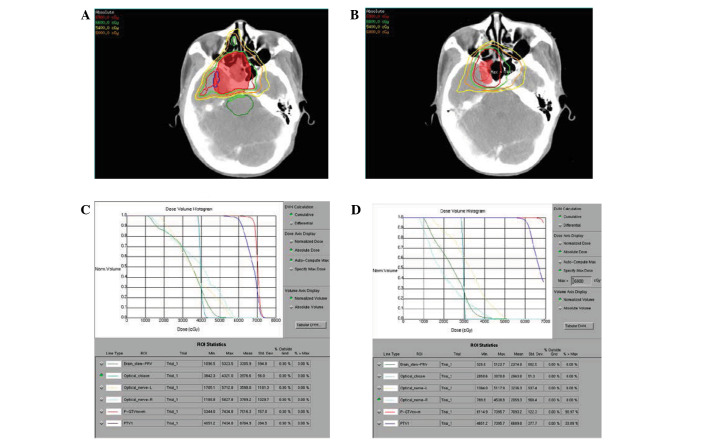
The intensity modulated radiation therapy (IMRT) change before and after interventional therapy. Isodose curve change of IMRT planning target volume before (A) and after (B) interventional therapy. Organ DVH change of IMRT planning target volume and organs at risk before (A) and after (B) interventional therapy. DVH, dose volume histogram.

**Table I.** The intensity modulated radiation therapy (IMRT) target area.

Target area name	Concept
GTVnx	Including imaging visible primary tumor site
GTVnd	Including imaging visible and confirmable metastatic cervical lymph nodes
GTVrn	Retropharyngeal lymph node
PGTVnx	(GTVnx + GTVrn) + extroverted 3–5 mm
PTVnd	Including (GTVnd + around high-risk lymph node) + extroverted 3–5 mm
CTV1	PGTVnx+ around high-risk lymph node
PTV1	CTV1 + extroverted 3–5 mm
CTV2	Including total neck lymph node drainage area
PTV2	CTV2 + extroverted 3–5 mm

**Table II.** Radiation dose.

Target area name	Single dose (Gy)	Total dose (Gy)	Number
PGTVnx	2.3	69	30
GTVnd	2.2	66	30
PTVnd	2.1	63	30
PTV1	2.0	60	30
PTV2	1.8	54	30

**Table III.** Tumor volume changes and locations before and after interventional therapy.

Case	Tumor volume (mm^2^)		Intracranial tumor volume (mm^2^)		Brain stem distance (mm)		Optic nerve distance (mm)		Optic chiasm distance (mm)	
				
	Before	After	Before	After	Before	After	Before	After	Before	After
1	50760	33072	10025	4361	2.1	3.5	3.8	4.7	4.0	5.2
2	67488	34920	16256	4782	1.2	4.0	3.2	4.5	3.8	6.5
3	61344	48396	15991	4330	1.5	3.1	3.1	4.2	3.8	5.2
4	35424	25200	6548	3356	2.6	3.6	5.5	5.5	4.2	5.0
5	43332	24312	6452	4233	3.0	3.8	3.8	4.3	4.1	5.4
6	32760	13872	5598	3945	3.0	3.7	4.8	5.0	4.8	5.3
7	47160	24288	8256	3476	2.5	3.3	5.3	5.8	5.5	6.3
8	69456	36132	13526	4621	1.2	2.8	3.3	4.8	4.4	6.0
9	39960	6780	6058	2935	2.3	4.6	4.8	5.2	5.5	6.5
10	23736	14964	7856	5046	3.2	3.5	5.2	5.6	4.8	5.2
11	50220	30360	6880	3821	2.2	2.9	5.3	5.5	5.2	6.1
12	27120	21792	7770	4438	2.8	3.2	4.7	6.1	5.1	6.9
Average	45730	26174	9268	4112	2.3	3.5	4.4	5.1	4.6	5.8

**Table IV.** Side effects during interventional chemotherapy.

Side effect	No. of cases	WHO toxicity grading			

		I	II	III	IV
Leucocyte decline	6	1	3	2	0
Hemoglobin decline	1	1	0	0	0
Platelet decline	4	3	1	0	0
Millet straw	3	3	0	0	0
transaminase rise					
Estomatitis	2	1	0	0	0
Nausea	8	5	2	1	0
Vomiting	5	3	2	0	0
Diarrhea	1	1	0	0	0

**Table V.** Dose change of organs at risk before and after interventional therapy.

Target area name	Before chemotherapy MDL (Gy)	Interventional chemotherapy MDL (Gy)	Drop ratio (%)
Brain stem	66.2	56.1	15.26
Optic chiasm	60.3	53.6	11.11
Optic nerve	46.5	40.2	13.55
Temporal lobe	72.6	66.1	8.95

MDL, maximum dose level.

**Table VI.** Side effects with intensity modulated radiation therapy (IMRT) concurrent chemotherapy.

Side effect	No. of cases	WHO toxicity grading			

		I	II	III	IV
Oral mucosa reaction	12	2	8	2	0
Nasal pharyngeal mucosa reaction	12	0	4	7	1
Skin reaction	12	8	3	1	0
